# Can Intranasal Insulin and Cholinergic Agonist Improve Post-Covid-19 Cognition Impairment?

**DOI:** 10.34172/apb.2024.017

**Published:** 2023-10-14

**Authors:** Amr Ahmed, Mahmoud El Kazzaz, Aml M. Brakat

**Affiliations:** ^1^Department of Public Health, Tuberculosis Program, First Health Cluster, Ministry of Health, Riyadh, Saudi Arabia.; ^2^Department of Chemistry and Biochemistry, Faculty of Science, Damietta University, Egypt.; ^3^Faculty of Medicine, Zagazig University, Egypt.

## Dear Editor,

 In December 2019, a novel coronavirus broke out as a pathogen in Wuhan, China, causing severe acute respiratory syndrome coronavirus 2 (SARS-CoV-2) that caused global healthcare crises and consumed health resources.^1^ In March 2020, the WHO declared that it was a pandemic.^2^ Classic COVID-19 symptoms include fever, cough, breathing difficulties, fatigue, diarrhea, sore throat, and loss of taste and smell.^3^

 Post-COVID-19 syndrome (PCS) is a persistent clinical manifestation that appears during or after suffering COVID-19, lasts for 12 weeks (about 3 months) or more, and cannot be explained by another diagnosis.^4^ Its prevalence ranges from 10% to 35% and may reach 85% among patients with a history of hospitalization.^5^ PCS affects multiple organs, including the brain. Cognitive impairment, disorientation, anxiety, headache, and sleep disorders are examples of neurologic symptoms. Herein, a published meta-analysis revealed memory impairment (27%, 18%-36%) and brain fog (32%, 9%-55%) in patients with PCS.^6^

 The exact theory of memory impairment is not determined. There are possible hypotheses such as direct CNS invasion. The spread could be hematogenous, but the olfactory neural pathway is the most reported entry.^7^ Blood-brain barrier disruption through activation of angiotensin-converting enzyme 2 (ACE-2) receptor expressed in both capillary and neuronal endothelium because of Transsynaptic spread from the olfactory bulb after intranasal exposure.^8^ This leads to a significant loss of gray matter in areas with high connectivity to the olfactory system.^9^

 We hypothesize that intranasal insulin can improve memory function and olfactory threshold in anosmic patients because Insulin signaling has been associated with olfactory function both directly, Insulin receptors are found in primary olfactory regions such as the olfactory bulb and indirectly, with a neuroprotective effect or regeneration of the olfactory mucosa.^10,11^

 Another theory is the dysfunction of the cholinergic anti-inflammatory pathway with acetylcholine receptor (AChR) antagonistic action. Cholinergic synapses have a precise role in the central cognition nervous system. muscarinic receptor subtypes have been implicated mainly in attention, arousal, and cognition; the nicotinic α7 receptor subtype is involved in working memory; whereas the α4β2 subtype affected attention tests. Nicotinic receptors have been linked with depression and anxiety modulation.^12^

 Alpha7 nicotinic acetylcholine receptor (α7-nAChR) activation which is mainly expressed by B cells, T cells, and macrophages, decreases proinflammatory cytokines formation as interleukin-1 (IL-1), interleukin-6 (IL-6), interleukin-8 (IL-8) and tumor necrosis factor-α (TNF-α) by nicotine or nicotinic agonists.^13,14^ Also, increased inflammatory cytokine may follow disruption of the polarization mechanism of M1 type macrophage through the antagonistic action on α7-nAChRs by SARS-CoV-2.^15^ This explains the cytokine storm and the hyperinflammatory syndrome seen in several COVID-19 patients. Supporting the hypothesis that nicotine and other cholinergic agonists have a protective role, there are observations that smokers are protected against SARS-CoV-2 hospitalization.^16–18^

 We can conclude that SARS-CoV-2 has anticholinergic action as scopolamine which is a muscarinic receptor antagonist.^19^ Scopolamine has been used as a sedative, but its usage is limited in past years as Scopolamine produces similar memory deficits seen in the elderly because blockade of central muscarinic receptors could induce a pattern of cognitive impairment.^20^

 Therefore, clinicians should be cautious about prescribing drugs with anticholinergic action such as scopolamine, benzodiazepines, opioids, tertiary amine tricyclic antidepressants, low-potency antipsychotics, benztropine, and diphenhydramine as these medications may cause or exacerbate cytokine storm and cognition impairment. Clinicians should consider these effects, especially in elderly patients because there is greater sensitivity to these treatments and their adverse effects, which may be exacerbated in cases of SARS-CoV-2 infection.^21^

## Competing Interests

 The authors declare no conflict of interest in this study.

## Ethical Approval

 Not applicable.

## Funding

 None.
